# The role of the early-life gut microbiome in childhood asthma

**DOI:** 10.1080/19490976.2025.2457489

**Published:** 2025-01-30

**Authors:** Ulrika Boulund, Jonathan Thorsen, Urvish Trivedi, Kaare Tranæs, Jie Jiang, Shiraz A. Shah, Jakob Stokholm

**Affiliations:** a Copenhagen Prospective Studies on Asthma in Childhood, Copenhagen University Hospital, Herlev-Gentofte, Gentofte, Denmark; b Section of Microbiology, Department of Biology, University of Copenhagen, Copenhagen, Denmark; c Department of Food Science, University of Copenhagen, Copenhagen, Denmark

**Keywords:** Childhood asthma, gut microbiome, early-life, virome

## Abstract

Asthma is a chronic disease affecting millions of children worldwide, and in severe cases requires hospitalization. The etiology of asthma is multifactorial, caused by both genetic and environmental factors. In recent years, the role of the early-life gut microbiome in relation to asthma has become apparent, supported by an increasing number of population studies, *in vivo* research, and intervention trials. Numerous early-life factors, which for decades have been associated with the risk of developing childhood asthma, are now being linked to the disease through alterations of the gut microbiome. These factors include cesarean birth, antibiotic use, breastfeeding, and having siblings or pets, among others. Association studies have highlighted several specific microbes that are altered in children developing asthma, but these can vary between studies and disease phenotype. This demonstrates the importance of the gut microbial ecosystem in asthma, and the necessity of well-designed studies to validate the underlying mechanisms and guide future clinical applications. In this review, we examine the current literature on the role of the gut microbiome in childhood asthma and identify research gaps to allow for future microbial-focused therapeutic applications in asthma.

## Introduction

Asthma is the most common chronic childhood disease, affecting approximately 23 million children globally.^1^ It is an inflammatory disease that often develops early in life and is characterized by symptoms such as wheezing and shortness of breath, with severe cases requiring hospitalization.^2^ While the exact etiology of asthma is unclear, genetic risk factors,^3–8^ birth mode,^9,10^ early life respiratory infections,^11^ and antibiotic exposure,^12^ as well as exposure to environmental factors, such as air pollution^13–15^ and dust^16,17^ are known risk factors. Several studies implicate the early-life gut microbiome in the development of asthma^10,18–28^ and other related diseases such as allergy^29^ or atopic dermatitis,^20^ which share several risk factors and pathophysiological mechanisms.^30^

The human gut microbiome is established shortly after birth, and its composition is influenced by several factors such as delivery mode,^31–33^ diet,^34–36^ medication,^33,37^ the home environment,^38^ and to a minor degree genetics.^39–41^ Gut microbes regulate numerous functions in the human host, such as training the immune system,^42–44^ protecting against pathogenic infections,^45–47^ producing vitamins^48^ and short-chain fatty acids (SCFAs),^49^ as well as breaking down food to allow for uptake of essential nutrients.^50–52^ The gut-lung axis represents the relationship between the two compartments, and how they affect one another.^53^ Several proposed mechanisms exist for this link, such as the activity of gut microbes via the immune system^54–58^ or the effect of microbially derived metabolites on the immune status of the airways.^59–61^

This review focuses on the relationship between the early-life gut microbiome, specifically bacteria and viruses, and childhood asthma. With the complexities of childhood asthma, such as disease types, etiology, and pathophysiology, alongside the intricacies of the gut microbiome ecology consisting of thousands of different microbes, this area of research holds great potential for clinical applications.

## Characteristics of childhood asthma

Asthma is an inflammatory disease in the airways usually characterized by attacks of excessive bronchoconstriction and airway hyperresponsiveness. This leads to attacks of airflow obstruction, causing symptoms such as wheezing, shortness of breath, chest tightness, and coughing, which greatly impact quality of life. The disease usually originates in childhood and changes its appearance throughout life, which makes age an extremely important variable, alongside precise phenotypic characterization, to consider when evaluating asthma as a study endpoint. Depending on the age and the clinical presentation of the child, episodes of asthma/wheeze in the preschool years may be labeled as “wheezing illness”, “preschool wheeze” or “preschool asthma”.^62^ Preschool wheezing is very common in the Global North, with around one-third of parents reporting symptoms before the age of three years; however, almost 60% of these children are in remission at age 6 years.^63^ Such preschool wheezing disorders are heterogeneous and may be influenced by specific risk factors, including host genetics, susceptibility, and environmental exposures.^62^

The current definitions of asthma are not fit for performing a precise diagnosis in preschool children, as they rely on objective measurements (e.g. forced expiratory volume in 1 second, forced vital capacity, and fractional exhaled nitric oxide) seldom available in this age group.^64^ Phenotypically asthma can be classified into allergic/atopic asthma and non-allergic/atopic asthma based on the presence of atopy. Allergic/atopic asthma is characterized by concurrent allergic sensitization (positive skin prick test or elevated specific immunoglobulin E (IgE)) and atopic disease (allergic rhinitis and/or atopic dermatitis), whereas non-allergic/atopic asthma represents asthma without these comorbidities. Asthma characteristics also differ by age, in adults and older children asthma is usually characterized by eosinophilic inflammation of the airways, while this is rarely the case among younger children. Consequently, research has sought to evaluate the underlying functional subtypes (endotypes) of asthma, of which the T2-high (eosinophilic) endotype is the most common and well-characterized. This endotype is distinguished by a high degree of atopy, elevated eosinophil levels in both sputum and blood, increased levels of Type 2 cytokines (IL-4, IL-5, and IL-13),^65^ and evidence of airway remodeling^66^ (e.g. loss of epithelial integrity, increased smooth muscle mass, goblet cell and submucosal gland enlargement, and neovascularization). Eosinophilic inflammation is often absent among young children, representing the non-atopic and non-eosinophilic (T2-low) endotype.^63,67^ Lower airway neutrophilia has also been described among preschool children with asthmatic symptoms, but whether the neutrophils here act proinflammatory, protective or just secondary to bacterial colonization/infection remains unknown.^68^ Effects of early environmental and microbial exposures may depend on the child’s genetic risk for asthma,^69–71^ a phenomenon known as gene-environment interaction, or susceptibility based on maternal asthma status.^72^

These factors are all extremely important to keep in mind when doing association studies in children, validation studies *in vivo*, and clinical intervention trials. Studies investigating asthma may in fact analyze different facets of the disease, depending on the age of the participant, the age at asthma diagnosis, whether it is allergic or non-allergic asthma, a T2 high or low phenotype, and whether a genetic susceptibility exists. Preschool asthma is not a “one size fits all” diagnosis. Many studies on the gut microbiome in relation to asthma are conducted in preschool children without taking the child’s genetic risk, phenotype of asthma, or disease trajectory and remission into account, which hampers comparability across studies.

## Early-life risk factors of asthma mediated through the gut microbiome

**Figure 1.**
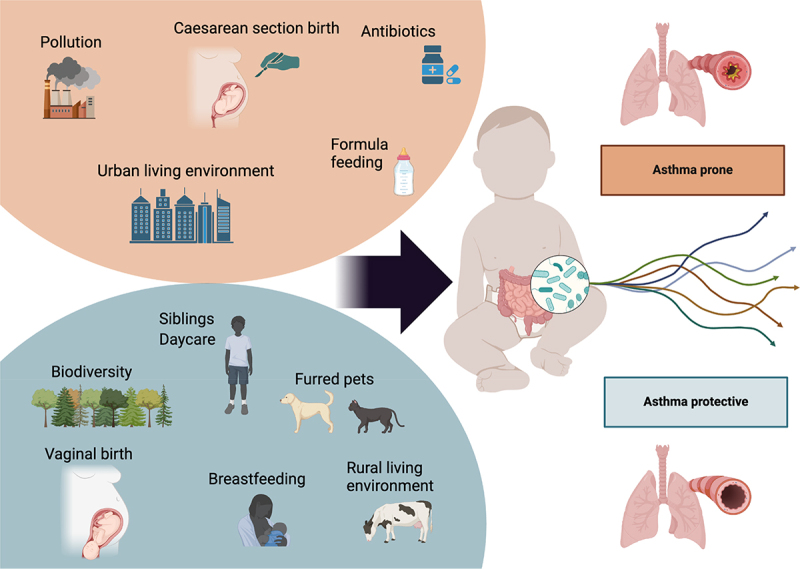
Environmental factors, gut microbiome development trajectories and childhood asthma.There are several factors that may influence the trajectory of an individual child and their risk of developing asthma, many of which are microbiome-mediated.

The incidence of asthma has increased in industrialized countries together with other non-communicable diseases over half a century,^73^ however it seems to have plateaued in recent decades.^74^ This increase in risk cannot be explained by changing genetics, which makes the early-life environment an obvious place of investigation. The study of the early-life human microbiome in relation to asthma and related immune-mediated disorders originates from the many epidemiological studies on the relevant early-life risk factors. Several environmental factors have been suggested to affect asthma risk, and many of these could exert their effects via the gut microbiome. Figure 1 illustrates some factors that can influence a child’s trajectory to an increased or decreased risk of developing asthma, many potentially mediated by the gut microbiome.

A newborn is born sterile^75^ and is in all likelihood exposed to the very first microorganisms during delivery.^75,76^ Being born by cesarean section is one of the strongest and most consistent environmental risk factors for asthma development.^77^ In recent years, it has been shown that especially the gut microbiome is affected when a child is born by cesarean section, a perturbation which in some cases may be long-term. Cesarean-born children have lower alpha diversity, as well as a lower relative abundance of the phyla Bacteroidetes and Actinobacteria while an increased abundance of Firmicutes and Proteobacteria. Additionally, cesarean-born children whose gut microbiome do not resemble the gut microbiome of vaginally born children within the first year of life are at much higher risk of developing asthma as compared to the children in which the initial microbial perturbation resolves.^10^ This suggests new possibilities for disease prevention by targeting early-life microbiome disruptors such as cesarean section.^78^ Antibiotics impact the gut microbiome, and it is recommended practice to give antibiotics during a cesarean section. To evaluate whether the use of intrapartum antibiotics could be the cause of the effects seen in cesarean section, antibiotic use in vaginally born children has been studied. This demonstrated that the gut microbiome composition of vaginally born children exposed to intrapartum antibiotics was intermediate between that of cesarean-born children and vaginally born children not exposed to intrapartum antibiotics.^10^ Thus, antibiotics may explain some, but not all, of the effects of cesarean section on a child’s microbiome. Antibiotic use prenatally or in early life has also independently been associated with increased asthma risk.^79,80,^ In children, such association has been proposed to go through effects on the gut microbiome,^81,^ however, the pregnancy association has been suggested to be primarily conferred by the mother’s propensity for infections rather than as a modulation of the gut microbiome.^82,^

Breastfeeding can modulate the gut microbiome in early life through multiple mechanisms,^83^ and several studies have suggested a protective effect of breastfeeding on the risk of developing asthma.^84–87^ Interestingly, a study in the VDAART cohort published in 2023 demonstrated that a large proportion of the protective effect of breastfeeding on asthma is mediated by gut microbiome maturity,^88^ suggesting a role for the gut microbiome in this relationship. Likewise, it has been observed that breastfeeding can potentially help alleviate some of the microbial alterations among cesarean-born children.^81,89^ Part of these effects could be due to the modulating effects of human milk oligosaccharides found in breastmilk on the gut microbiome.^90^

The original hygiene hypothesis^91^ suggested that growing up with older children in the home led to protection from developing allergies. Siblings (especially with a short age gap) constitute a major factor in shaping the early-life gut microbiome by transferring diverse microbes (both commensal microbes and pathogens),^92,93^ and promote an accelerated gut microbiome maturation^19,94^ as well as recovery from microbial perturbation from cesarean section.^10^ The exposure to a greater microbial diversity by older siblings is believed to aid immune system development by training the infant’s immune system to differentiate between harmful and benign stimuli and thus promoting tolerance, which reduces the risk of atopic disease development. Moreover, changes in parental behavior regarding hygiene and diet may further enhance the sibling effect. It was recently shown that gut microbiome maturation was a key mediating factor in the link between siblings and protection from food allergy.^95^ Akin to having siblings, many children are exposed to other children through daycare attendance. There have been several studies reporting a reduced risk of asthma in children who attend daycare,^96,97^ potentially due to a sibling-like effect, however some report an increased risk,^98^ which may be due to a higher risk of early-life infections.^99^ While the hygiene hypothesis has been interpreted as children growing up in environments with lower microbial richness and load may be at higher risk of disease due to limited exposure to pathogens, it has later been nuanced through the “old friends hypothesis” highlighting the importance of exposure to commensal bacteria with which humans have co-evolved, playing a key role in immune system development.^100^

The “biodiversity hypothesis” argues further that diverse external environmental influences are also critical for shaping the developing immune system.^101^ Such influences have been exemplified in the protection from asthma by living in close contact to livestock in farming environments (contact with livestock, animal feed, hay, grain and unprocessed cow’s milk via inhalation or ingestion)^102^ and by growing up in a rural environment which typically exhibit a richer microbial diversity compared to urban environments^38^ It is important to note that urban vs. rural living and air pollution are strong risk factors for childhood asthma, and these are strongly correlated which makes the disentangling of specific effects complex to investigate.^103^ These exposures have been shown to impact both the external^104^ and inhabiting^38^ microbiome, which can have an effect on both innate and adaptive immune mechanisms to reduce inappropriate inflammatory response. In a similar manner, pets have been shown to influence the microbiome of the people in the household through transfer of diverse microbes^105,106^ which can affect immune regulation. Growing up with a dog^107^or a cat^70,^ in the home has been shown to reduce the asthma risk in individuals who carry the strongest known genetic variant for childhood asthma, suggesting gene-environment interactions affecting the asthma risk. This is one example of how variations in the studied population or diagnosis can explain why studies often present discrepancies in directionality of exposures.

## Human studies on the gut microbiome and immune development in childhood asthma

Since the emergence of the hygiene hypothesis,^91^ microbial exposure and microbial diversity have been linked with allergic diseases, including asthma. Historically, single bacterium species characterized by culturing or qPCR has been evaluated in relation to later development of allergic diseases.^20,29^ Since then, studies have applied methods such as gel electrophoresis to assess the gut microbiome as a whole in birth cohorts.^18^ Newer molecular techniques, including 16S rRNA gene amplicon sequencing and metagenomic shotgun sequencing, have made it possible to deeply characterize the taxonomic and functional repertoire and composition of the microbiome. This can be applied to fecal samples collected from longitudinal birth cohorts with prospective asthma diagnoses to directly assess the association between the early-life gut microbiome and childhood asthma. While early efforts focused on the notion of diversity as a hallmark of a healthy gut microbiome, recent longitudinal investigations have charted the natural development of the microbiome as a nonlinear process involving the succession of many species.^108,109^ The microbiome is first established with early colonizers able to thrive in a low-complexity environment, then impacted by breastfeeding and solid food introduction triggering shifts in nutrient availability and aiding immigration of novel species into the community. Eventually, specialized species emerge with multiple ecological dependencies and complex community structures in the microbiome.^109–111^ While this process steadily increases diversity throughout the first year(s) of an infant’s life, the notion of maturation of the infant gut microbiome has also emerged as an important concept in the field.^112^ As such, an infant’s chronological age and their gut microbiome composition can be considered to develop along parallel trajectories, and an appropriate microbiome maturity relative to an infant’s age is regarded as an indicator of healthy development^19^.

Among larger prospective birth cohort studies, a study of 319 children from the Canadian CHILD birth cohort was the first to report data from human subjects associating sequence-based characterizations of the human gut microbiome with a later asthma phenotype.^113^ In this study, the authors reported reduced levels of *Faecalibacterium, Lachnospira*, *Veillonella*, and *Rothia* (FLVR) at 3 months in children with atopy and wheeze at 1-year of age. This phenotype was associated with a higher risk of later asthma development. The study also reported reduced levels of short-chain fatty acids and other metabolites at 3 months of age in these children and showed that supplementation with FLVR-species attenuated airway inflammation in mice inoculated with fecal samples from the atopic wheeze children. A study of 298 neonates from the Wayne County birth cohort found that gut microbiome states at 0.5 to 4.5 months associated predominantly multisensitized atopy at age 2 and parent-reported doctor-diagnosed asthma at age 4.^21^ The high-risk gut microbiome state was characterized by depletion of *Bifidobacterium, Lactobacillus, Faecalibacterium* and *Akkermansia*. Further, the high-risk state was associated with pro-inflammatory metabolites, including 12,13-dihydroxy-9Z-octadecenoic acid (12,13-DiHOME), that induced differential activation of CD4^+^ T cells.

A study of 690 children from the COPSAC_2010_ mother-child cohort utilized the concept of microbiome age^40^ to link delayed maturation of the gut microbiome at 1 year of age with the risk of doctor-diagnosed asthma by age 5 years.^19^ Notably, this delayed maturation was accompanied by lower abundances of *Faecalibacterium*, *Bifidobacterium*, *Roseburia, Alistipes, Lachnospiraceae, Ruminococcus, Dialister*, and higher relative abundance of *Veillonella* and was only apparent in children born to mothers with a history of doctor-diagnosed asthma. The association of gut microbiome maturation and asthma was replicated and expanded in several following studies. In the PASTURE birth cohort (*n* = 930), of which 50% of the children grew up on family-run farms, associations between the gut microbiome at 2 and 12 months of age, and asthma development at age 6 were reported.^114^ Further, they replicated the association between gut microbiome maturation and asthma, and identified a mediation of the protective farm-effect,^115^ especially conferred by butyrate production. The taxa of interest that associated with both maturation and butyrate concentrations included *Roseburia* and *Faecalibacterium*. In an expanded analysis from the CHILD study (*n* = 589), the association between gut microbiome maturation and asthma was supplemented to also encompass allergic rhinitis, atopic dermatitis and food allergy.^116^ Important promoters of gut microbiome maturity were *Clostridium innocuum, Ruminococcus gnavus, Faecalibacterium prausnitzii, Anaerostipes hadrus* and *Blautia wexlerae*. Similarly, data from the Barwon Infant Study showed that gut microbiome maturation at 1 year was associated with having older siblings and inversely associated with food allergy at 1 year (*n* = 323).^95^ Additionally, in the US based VDAART cohort, they replicated that low gut microbiome maturity at age 1 year was a risk factor for asthma, while an early (3–6 months) high maturation was also a risk factor, pointing to a timely maturation being important.^88^

Another study from the CHILD cohort demonstrated that breastfeeding could ameliorate the antibiotic-induced disruption of the microbiome, thus reducing the subsequent risk of asthma.^81^ Importantly, the mitigating effect of breastfeeding appeared to be due to selective enrichment of *Bifidobacterium longum* subsp. *infantis* in infants who were breastfed while receiving antibiotics. This highlights how the gut microbiome interacts with various factors and events during early infancy, influencing immune development and shaping the risk of future diseases.

As we have presented here, numerous prospective association studies have revealed the potential role of the gut microbiome in the development of childhood asthma, identifying several candidate bacteria, consortia, and the overall microbiome maturity, as important determinants.^19,21,81,88,113,114,116^ These studies are summarized in Table 1. Due to the clinically relevant designs, robust asthma diagnoses, and often long follow-up, these reports allow for the investigation of the development of the gut microbiome and asthma over time. Furthermore, they reflect realistic clinical manifestations of the disease and the heterogeneity and complexity of the gut microbiome across geography. These studies provide invaluable insight and are an important contribution to the design and directions of further research. Despite this, the findings are sometimes conflicting, possibly due to e.g. variation in study design, technical methodology, sample size, or geographical location (reflecting microbiome variations). Furthermore, these studies inherently lack causal conclusions and mechanistic insights of disease development. Therefore, *in vivo* studies allowing for analysis of the underlying mechanisms and discerning causal links from associations are essential. Emerging evidence indicates that the infant microbiome may be susceptible to microbiome-based interventions in early life^78,117^ which is a promising avenue for which many types of interventions could be envisaged. Whether such interventions could lead to asthma prevention is a research question of very high priority, and we cover several studies investigating this in a section below.

**Table 1.** Key studies on the relationship between the infant gut microbiome and childhood asthma. 16S: 16S rRNA amplicon sequencing. ISAAC: international study of asthma and allergies in childhood.

Study	N	Main clinical outcome(s)	Main microbiome factor(s) associated with decreased/increased asthma risk	Additional notes/findings
Arrieta et al. 2015^113^	319	1-year atopic wheeze	3 months *Faecalibacterium*, *Lachnospira*, *Veillonella*, and *Rothia* (FLVR)	16S; FLVR given to mice attenuates airway inflammation
Fujimura et al. 2016^21^	298	2-year multi-sensitised atopy; 4-year parent-reported doctor-diagnosed asthma	1–11 months microbiome clusters - asthma-associated cluster depleted of *Bifidobacterium*, *Akkermansia* and *Faecalibacterium*	16S; CD4 experiment
Stokholm et al. 2018^19^	690	5-year investigator-diagnosed asthma	High 1-year microbiome-by-age z-score (MAZ), many taxa including *Faecalibacterium*, *Bifidobacterium*, *Roseburia*, *Alistipes*, Ruminococcus	16S; Stronger in mothers with asthma
Depner et al. 2020^114^	930	6-year doctor diagnosed and/or parent reported asthma	12 months Estimated microbiome age; *Roseburia* and *Faecalibacterium*	16S; Mediates farm effect & link with butyrate metabolism
Chen et al 2023^88^	463	3-year physician-diagnosed asthma or recurrent wheeze	3–6 months MAZ score 1-year MAZ score 3–6 months *Bacteroides*	16S; Mediation of breastfeeding protection, independent and additive with 17q12–21 genetic risk variants
Hoskinson et al. 2023^116^	589	5-year asthma based on doctor assessment and ISAAC questionnaire^118^	1-year microbiome-predicted age, driven by multiple taxa. *Anaerostipes hadrus*, *Fusicatenibacter saccharivorans, Eubacterium hallii, Blautia wexlerae*	Metagenomics; Shared association with allergic rhinitis, atopic dermatitis, food allergy
Dai et al. 2023^81^	1,338	5-year investigator-diagnosed asthma	Breastfeeding mitigates antibiotics-associated asthma risk via 1-year microbiome enrichment of Bifidobacterium longum subsp. infantis	Metagenomics;

## Manipulation of the gut microbiome composition and the role of the gut virome in childhood asthma

While the link between asthma and gut bacteria is well established, the role of intestinal viruses, particularly bacteriophages (phages), is less known.^119^ Phages infect bacterial cells and thus affect the human host mainly by regulating the bacterial community. Phage therapy utilizes virulent phages to kill targeted pathogenic bacteria and has a long scientific and clinical history.^120,121^ However, this methodology relies on specific knowledge of the causal bacteria involved in a disease, and is not capable of implementing overall compositional changes. Fecal microbial transplantation (FMT) is an approach to improve the overall gut microbiome composition through the use of feces from a healthy donor and is mainly utilized in *Clostridium difficile* infections,^122^ but has also been applied to other diseases.^123^ However, reports of severe adverse events, such as the transplantation of pathogenic or antibiotic resistant bacteria,^123^ as well as the risk of transferring virulence genes or parasitic microorganisms,^124^ demonstrate the risks of this procedure and the need for novel advances. Different methods of fecal virome transplantation (FVT) and similar approaches (e.g. fecal filtrate transfer (FFT)) have been tested as alternatives to FMTs, circumventing many of the risks of bacterial transfer while utilizing the bacteriome-modulating potential of bacteriophages.^125^ An FFT contains, besides viruses, also metabolites, peptides, and bacterial DNA, while an FVT is processed with an additional filtering step that allows for the discarding of metabolites, peptides etc., and thus contains in essence only viruses.^126–128^ Interestingly, the bacterial composition of FFT recipients has been shown to resemble that of the donor.^129^ As the FFT contains other compounds and bacterial DNA from the donor besides viruses, it can potentially stimulate the recipient’s microbiome through modulation of the immune system or the microbial activity. Current research is investigating how FVT can be further adjusted to reduce potential risks.^130^ However, several obstacles remain for both FMT and FVT, such as how to identify and define a healthy donor, whether combinations of donors, personalized donors, or related donors should be used, not to mention the costs associated with identifying appropriate donors.^131^ Additionally, the route of donation (capsules, colonoscopy, nasoduodenal tube) may play a role in the engraftment of the microbes and the success of the procedure.^132,133^ These factors must be considered and evaluated in future studies to instruct the possibility of clinical applications.

There is already some evidence of the effects of FVT in mouse models^134,135^ as well as in human trials on various diseases.^129,136–138^ A current clinical study is investigating FFT for the treatment of necrotizing enterocolitis (NEC), a poorly understood and life-threatening gastrointestinal disorder afflicting preterm infants, partly characterized by microbiome perturbation and bacterial overgrowth.^139^ Oro-gastric FFT administration prevented NEC in a preclinical piglet model, increased viral diversity, and reduced ileal mucosa Pseudomonadota (Proteobacteria) relative abundance. Finally, mother-to-infant FVT has recently been approved for experimental restoration of the microbiome perturbation associated with birth by cesarean section,^140^ as an extension of previous evidence demonstrating the normalizing effect of mother-to-infant FMT after cesarean section.^78^

In addition to the modulatory effect of phages on the bacterial community, emerging evidence suggests that phages can also affect non-bacterial cells directly.^141^ TLR9 activation has been demonstrated in dendritic mouse cells pulsed with phage antigens following exposure to phage particles containing single-stranded DNA genomes rich in CpG motifs^135,142^ and single-stranded DNA derived from M13 phages has been shown to activate serum interferon production in mice.^143^ In human cells, phage uptake occurs by endocytosis^144,145^ and once inside a cell, phages can affect cellular responses. A study demonstrated that human peripheral blood mononuclear cells infected with the *Pseudomonas aeruginosa Pf4* phage were prevented from phagocytosing and displayed altered antibacterial receptor and cytokine production, effectively preventing the clearing of *P. aeruginosa* from infected wounds.^144^ Although evidence of human TLR-phage interaction is absent, such activation is plausible, given the many structural similarities between phages and eukaryotic viruses.^146^

As opposed to gut bacteria, virome-disease association studies are still sparse. The virome comprises a small proportion of the overall gut microbiome. Therefore, removing bacterial cells and fragments before sequencing is an important step to reserve enough sequencing depth to fully estimate the diversity of the virome composition.

Another complexity is the virome dark matter problem,^147^ as viruses are more genetically diverse than bacteria and don’t have a specific marker gene such as the 16S rRNA gene for bacteria, and thus cannot be identified by traditional methods. Additionally, most human viruses don’t exist in databases, partially due to the high individual uniqueness within each sample. Recent studies have attempted to exhaustively resolve sample viral dark matter both through manual curation,^148^ by the use of marker genes,^149^ or by assembly of niche-specific viral databases.^150^ One such dark-matter-resolved infant gut virome data set^148^ uncovered an association between the fecal virome and the development of asthma in 647 one-year-old children from the COPSAC_2010_ cohort.^151^ The temperate virome composition was altered and exhibited a higher abundance in children who developed asthma by age five. Furthermore, several phage families infecting a variety of bacteria such as Clostridiales, Oscillospirales, and Bacteroidales, had a lower abundance in this group. Additionally, the association of the phages was partially independent from that of the bacteria, and revealed interactions with the TLR9 genotype of the child, suggesting that intestinal phages can directly influence the risk of childhood asthma. This is the only published paper on the role of the intestinal virome in childhood asthma; however, the results from this study are strengthened by similar findings of an independent association of phages with atopic dermatitis,^152^ a disease with several similarities to asthma.^30^ Together, these data hint at phages’ immunogenicity and potential in human disease in early life. Future research should seek to elucidate the mechanisms of direct human-phage interactions and focus on characterizing the virome dark matter in human studies to further delineate gut virome diversity. Apart from bacteriophages, the gut virome also contains a small fraction of eukaryotic viruses that infect human cells, many of which remain elusive in terms of their effect on the host. The eukaryotic virome can be influenced by various factors and could be of importance in immune system development in early life.^153–155^

## Mechanistic insights into gut microbiome contributions to asthma pathogenesis

Asthma, as observed in humans, is a complex disease that is difficult to recapitulate in animal models. However, certain animals exhibit asthma-like conditions, such as eosinophilic bronchitis in cats and heaves in horses.^156,157^ Over the past few decades, a variety of animals have been used to study asthma, including *Drosophila melanogaster* (fruit flies), guinea pigs, rats, dogs, swine, cattle, sheep, horses, and primates.^158–163^ While each species offers unique advantages and insights into disease-related mechanisms, mice have become the most widely used species in asthma research due to their ease of breeding, handling, and genetic manipulation.^164^

Murine models, especially the BALB/c, C57BL/6 and A/J strains, have been instrumental in advancing our understanding of atopic disease and the role of Th2-mediated immune responses in asthma.^164,165^ However, it is important to note that mice do not spontaneously develop an asthma-like phenotype; instead, the disease must be artificially induced. Both acute and chronic models of asthma have been developed in mice, each with its own strengths and limitations.^166,167^ Acute mouse models have successfully reproduced many features of asthma, including elevated levels of serum IgE, airway inflammation, goblet cell hyperplasia, and airway hyperresponsiveness. However, these models differ from human asthma in that the pattern and distribution of pulmonary inflammation are not identical, and airway inflammation tends to resolve within a few weeks after the final allergen challenge.^168,169^ Chronic mouse models, on the other hand, more closely replicate human asthma, particularly in terms of airway remodeling and sustained inflammation. These models involve repeated allergen exposure, leading to persistent airway hyperresponsiveness and lung inflammation, which better mirrors the chronic nature of asthma in humans.^170–173^

The study of allergic asthma in animal models involves a two-phase process: sensitization and challenge. Sensitization is the process where an animal is first exposed to an allergen, priming the immune system to recognize it, leading to the production of specific IgE antibodies that bind to mast cells and basophils.^174,175^ As a result, the immune system becomes more responsive to future allergen exposures. The challenge phase involves re-exposing the sensitized animal to the allergen, triggering an immune response with the activation of mast cells and basophils, leading to bronchospasm, airway edema, and mucus secretion typical of asthma.^175^ A late-phase response may follow, marked by the recruitment of eosinophils and other leukocytes, resulting in chronic eosinophilic inflammation, a hallmark of adult asthma. A wide range of allergens have been used to study asthma, with ovalbumin being commonly used due to its availability and strong allergic response induction,^176^ though it is not a natural human asthma allergen. To address this, researchers use house dust mites, which are relevant human asthma triggers and more closely resembles the natural route of allergen exposure in humans. House dust mite allergens are effective in inducing allergic inflammation due to their enzymatic activity and ability to activate innate immune cells through the Dectin-2 receptor.^177,178^

Murine models have been instrumental in linking the gut microbiome to asthma by demonstrating how early-life microbiome alterations influence immune responses and asthma susceptibility. In a study, where mice were exposed to antibiotics (i.e. azithromycin or amoxicillin) during early life, the resulting gut microbiome perturbations were found to heighten allergic responses upon later exposure to the house dust mite allergens.^179^ Specifically, mice exposed to azithromycin showed increased levels of IgE and IL-13, key indicators of allergic asthma, along with altered gut microbial composition, including reduced diversity and changes in key taxa like *Lachnospiraceae* and *Muribaculaceae*. When germ-free mice were colonized with microbiome from antibiotic-exposed mice, their offspring developed heightened immune responses and asthma-like symptoms, illustrating that early-life gut microbiome composition can heighten allergen-driven Th2/Th17 immune pathways and airway responses in an age-dependent manner. Another study established a causal link between the early-life microbiome and asthma susceptibility using a murine model with humanized microbiome.^113^ Here, they colonized germ-free mice with fecal samples from human infants at high risk of asthma and observed increased airway inflammation in these mice. However, when the mice were supplemented with four specific bacterial genera—*Faecalibacterium*, *Lachnospira*, *Veillonella*, and *Rothia* (FLVR) – which were found to be deficient in infants developing atopic wheeze, the mice showed a significant reduction in lung inflammation. These and other studies show that specific gut bacteria, when altered early in life, can influence immune development in ways that increase asthma susceptibility^180,181^ highlighting the potential for microbiome-based interventions in early life to reduce asthma risk. However, as previously discussed, children rarely suffer from the typical Th2-driven allergic phenotype, characterizing most adults with asthma. This can be a challenge and needs to be acknowledged, when deciding to make a translational mouse model for investigating gut microbiome and asthma in children.

Microbial-derived metabolites, such as SCFAs and tryptophan-derived metabolites, also influence pathways critical to asthma pathogenesis. SCFAs, particularly acetate, propionate, and butyrate, are produced by gut bacteria through fermentation of dietary fibers and bind to G-protein-coupled receptors (GPR41, GPR43, GPR109A), promoting the differentiation of regulatory T cells and inhibiting eosinophilic recruitment, suppressing Th2-driven allergic inflammation in asthma.^182,183^ SCFAs enhance Treg differentiation by inhibiting histone deacetylase,^183–186^ leading to epigenetic changes that upregulate immune-regulating genes, including those involved in the production of interferon-gamma (IFN-γ). This shift toward a Th1 immune response helps balance the Th1/Th2 axis, reducing the allergic inflammation. SCFAs also downregulate the expression of adhesion molecules on the endothelium, reducing eosinophil trafficking into the airways.^187^ In murine models, SCFA supplementation has been shown to reduce allergic airway inflammation, and human studies have found that lower levels of fecal SCFAs in infancy are associated with an increased risk of asthma later in life.^61,113,188,189^ Additionally, SCFAs enhance mucosal immunity by stimulating the production of IgA, which helps protect against allergens and pathogens in the airway.^190^

Tryptophan metabolites, primarily those derived from gut microbial metabolism, act through the aryl hydrocarbon receptor (AhR) and indoleamine 2,3-dioxygenase (IDO) pathways. AhR activation by metabolites such as indole-3-acetate, indole-3-aldehyde, and tryptamine enhances immune tolerance by promoting Treg differentiation and suppressing pro-inflammatory Th2 cytokine production, particularly IL-5, IL-13, and IL-33, all of which are central to the allergic responses observed in asthma.^191–194^ AhR also modulates innate lymphoid cells (ILC3), leading to IL-22 production,^194^ which enhances epithelial barrier integrity. Concurrently, the IDO pathway metabolizes tryptophan into kynurenine, which inhibits T cell proliferation by reducing tryptophan availability and induces regulatory T cells through kynurenine receptor signaling. Importantly, IDO expression is regulated by interferon-gamma (IFN-γ), a cytokine critical in antiviral and antibacterial defense^195,196^ which can further dampen inflammatory responses in asthma by modulating Th1/Th2 balance. IFN-γ induces IDO in antigen-presenting cells, creating an immune-tolerant environment that suppresses allergic airway inflammation. These mechanisms highlight the multifaceted role of microbial-derived metabolites in modulating immune responses and reducing the risk of asthma through both systemic and local effects.

A new avenue that may bridge part of the gap between *in vivo* and human studies to aid in our understanding of the relationship between the gut microbiome and childhood asthma is gut organoids.^197^ There are several hurdles to overcome, such as the lack of immune cells which may be of high importance in the gut, alongside the lack of innervation and blood vessels.^198^ Furthermore, organoids demonstrate a limited cell diversity and cannot model multiorgan pathologies, which is of high importance when studying the relationship between the gut microbiome and asthma.^198^ However, further advances that allow for an increased complexity of organoids will likely aid in analyzing the effect of consortia of bacteria and their effect on the intestines.

Utilising model systems and organisms allows us to delve deeper into the identified associations between gut bacteria and asthma to disentangle the biological processes. However, model organisms or systems can never fully capture the heterogeneity of either asthma or the gut microbiome. Mouse models rely on induced asthma phenotypes, with slight differences in the representation and immune mechanism of the disease, similarly the gut microbiome of laboratory mice is vastly different from that of humans, both in terms of diversity, taxa, and function.^199^ Thus, to fully bridge the gap between human association studies and *in vivo* findings, human intervention studies are necessary.

## Clinical trials on pre- and probiotics in childhood asthma

During the first year of life, the gut microbiome is very dynamic and this period is often referred to as a ‘window of opportunity’ because of the potential for preventive interventions, where bacterial strains introduced have the potential of establishing themselves in the gut. One way to translate the findings on the relationship between the gut microbiome and asthma to the clinical setting is intervention trials with probiotic bacteria. Probiotic bacteria are defined as bacteria that, in sufficient quantities, confer a beneficial effect to the host.^200^ Based on previous findings, several potential probiotics have been put forward, e.g., members of the *Bifidobacterium*, *Lactobacillus*, *Lacticaseibacillus*, *Ligilactobacillus*, and *Lactococcus* genera.

The first systematic review that investigated the effect of probiotics on asthma covered four RCTs and was published in 2008,^201^ and concluded that none of the studies reported significant findings. In 2013, Elazab et al.^202^ published a systematic review investigating the administration of probiotics in early life on atopy. They evaluated 14 studies from 10 RCTs focused on asthma. They did not find a significant reduction of asthma or wheeze, as well as no evidence of effect in subgroup analyses based on age, treatment length, follow-up duration, probiotic strain, dose administered or outcome definition. In another systematic review, published in 2013 by Azad et al.,^203^ they analyzed 19 RCTs with probiotic supplementation during pregnancy or infancy on asthma. The authors noted that none of the studies were powered to detect asthma as the primary outcome, and the median follow-up was only 24 months. Overall, there was no significant evidence to suggest that probiotic supplementation was protective of asthma.

In 2019, Wei et al.^204^ published a systematic review of 27 studies on probiotics in asthma. The probiotics were given either prenatally, postnatally or as a combination of both, in some cases combined with a prebiotic fiber which acts as a food source for the gut bacteria. The studies were mainly conducted in populations with high infant asthma risk based on family history. The authors concluded that there was no significant effect of probiotics supplementation on asthma, nor any significant effects based on subgroup analyses regarding the timing of intervention, asthma risk, prevention regimen, probiotic organism, intervention duration, or follow-up duration.

Later in 2019 Du et al.^205^ investigated 16 RCTs, with a majority of participants with high asthma risk, and reported no significant effect of probiotic supplementation on asthma development. However, a subgroup analysis revealed that supplementing with *L. rhamnosus* specifically decreased the risk of developing asthma compared to placebo.

More recently, in 2022, Uwaezuoke et al.^206^ investigated three RCTs of probiotics in asthma. The probiotics (with or without prebiotic fiber) were given either postnatally or to older children. In summary, Cabana et al. found that *L. rhamnosus* reduced incidence of asthma by age 5; Huang et al. found that *L. paracasei* or *L. fermentum* or their combination improved clinical asthma outcomes; Drago et al. found that *Ligilactobacillus salivarius* and *B. breve* reduced frequency of asthma exacerbations. Uwaezuoke at al. did not conduct a quantitative analysis of these studies.

**Figure 2.**
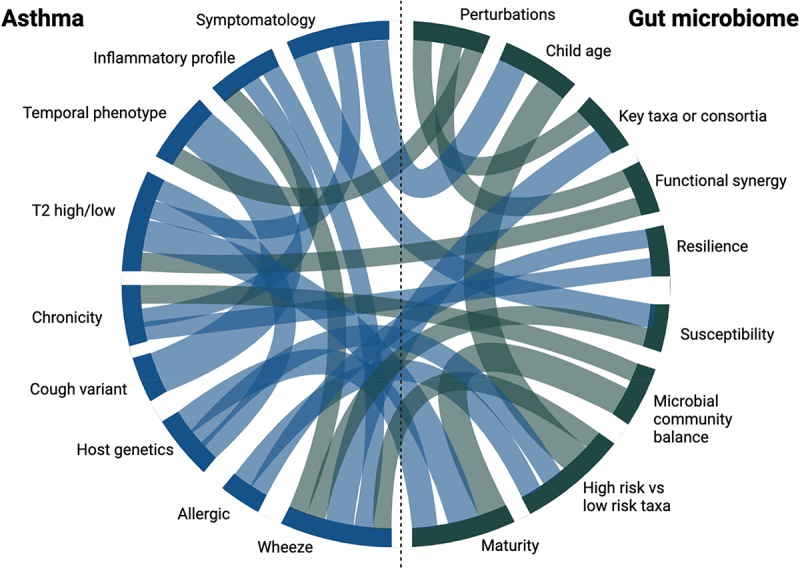
The complex facets of childhood asthma and the gut microbiome. Childhood asthma and the gut microbiome are complex entities with multiple facets that must be considered when studying their relationship. Heterogeneity in study design and findings hampers our understanding of the link between the gut microbiome and asthma. This figure was generated using hypothetical data.

In conclusion, there have been many RCTs investigating the efficacy of probiotics on childhood asthma, with some suggesting promising results; however, when analyzed in systematic reviews, the majority report no significant effect. Table 2 provides a summary of published meta-analyses investigating the effect of probiotic supplementation on asthma. The reasons for these discrepant findings may be that the complex facets of both asthma and the gut microbiome that we have presented in this review are not adequately addressed or considered. We present an illustration of some of these complex facets in Figure 2. Many studies had modest sample sizes and were not appropriately powered to model asthma as the outcome. Furthermore, the studies were sometimes conducted in general infant populations, without considering, e.g. age of diagnosis, genetic risk, or environmental factors, though some were conducted in high-risk populations based on family history. Additionally, the clinical definition of asthma varied widely between studies, leading to discrepancies in the asthma phenotypes studied (allergic/non-allergic, T2 high/low, wheeze, transient or persistent asthma, follow-up duration, or other asthma-related clinical characteristics). In regard to the probiotic intervention, the majority of the studies investigated single or simple combinations of existing probiotic strains, occasionally with prebiotics, but did not encompass the complexity of the gut microbiome or consider consortia of bacteria that have been found protective in prospective studies, detailed above.

**Table 2.** Overview of published meta-analyses investigating the effect of probiotic supplementation on asthma.

Meta analysis	Number of studies (unique studies)	Number of participants	Probiotic	Intervention duration	Follow-up duration	Main result
Vliagoftis et al. 2008^201^	4 (4 unique)	257	*E. faecalis*, *L. acidophilus*, *L. casei*, *L. rhamnosus*	4 weeks to 1 year	22 weeks to 1 year	No significant effect
Elazab et al. 2013^202^	14 (1 unique)	4,894	*B. lactis*, *L. acidophilus*, *L. casei*, *L. gasseri*, *L. paracasei*, *L. reuteri*, *L. rhamnosus*	1 month to 13.5 months	0–54 months	No significant effect
Azad et al. 2013^203^	19 (5 unique)	3,930 mothers and 4,766 infants	*B. bifidum*, *B. breve*, *B. lactis*, *B. longum*, *L. acidophilus*, *L. casei*, *L. fermentum*, *L. lactis*, *L. paracasei*, *L. reuteri*, *L. rhamnosus*, *L. salivarius*, *P. freudenreichii*	1 month to 25 months	4 months to 8 years	No significant effect
Wei et al. 2019^204^	27 (4 unique)	8,197	*B. bifidum*, *B. breve*, *B. lactis*, *B. longum*, *L. acidophilus*, *L. casei*, *L. paracasei*, *L. reuteri*, *L. rhamnosus*, *P. freudenreichii*	3 months to 24 months	6 months to 7 years	No significant effect
Du et al. 2019^205^	16 (7 unique)	5,264	*Bifidobacterium*, *L. rhamnosus*	3 months to 24 months	1 year to 15 years	Overall no significant effect, however subgroup analysis revealed that *L. rhamnosus* significantly decreased the risk of developing asthma compared to placebo
Uwaezuoke et al. 2022^206^	3 (2 unique)	1,320	*Bifidobacterium*, *B. breve*, *L. casei* *L. fermentum*, *L. paracasei*, *L. rhamnosus*, *L. salivarius,*	8 weeks to 6 months	8 weeks to 2 years	No overall quantitative analysis.

In this table only single probiotics are noted, though in some studies several combinations of probiotics were investigated, sometimes in combination with prebiotics. The column denoting the number of studies represents the number of studies included for meta analysis, also specifying how many studies were unique (not included in any of the earlier meta-analyses).

We previously mentioned that FMT and FVT from healthy donors could be considered for the prevention of asthma, as they are increasingly successfully used in other disease outcomes.^207^ One of the main benefits of FMTs or FVTs, as discussed above, is that they potentially modulate the entire microbiome, rather than introducing a single new bacterial species that was not present before. Interestingly, Durack et al. investigated the effect of *L. rhamnosus* supplementation to improve the overall gut microbial maturity,^117^ suggesting that overall microbial effects are in fact possible even with simple probiotic interventions. It has been demonstrated in numerous studies how important environmental factors are for the development of asthma,^103,208–210^ and some researchers are investigating the potential of biodiversity exposure to modulate the gut microbiome composition in an effort to alleviate asthma.^211^ Additionally, the timing of the intervention (prenatal, first week/months/years of life) is essential, as some probiotics may be more effective early or late in childhood, potentially dependent on factors such as delivery mode, microbiome maturation, breastfeeding, pets, or siblings. Moreover, the interval, duration, combination with a prebiotic, and method of the supplementation (capsules, drops, dairy products) was heterogeneous between the studies, and it is unclear which approach yields the best engraftment or clinical effect. It is believed that combining the probiotics with a prebiotic fiber may provide a beneficial environment for the probiotic strain and allow for it to be retained longer to yield an effect, however there are discordant findings on this.^212,213^ It is also possible that prebiotics alone may confer beneficial effects and less risks, acting as a fuel source for resident healthy gut microbes to thrive and outcompete pathogenic bacteria,^214^ improve gut barrier integrity^215,216^ produce short-chain fatty acids,^217,218^ and decrease intestinal pH promoting a beneficial microbiome composition.^219–221^ It is still unclear and intensely researched what factors influence the engraftment of different bacteria, and recent FMT studies suggest that permanent engraftment may not even be necessary to yield clinical effects.^132,133^

## Conclusion and future directions

**Figure 3.**
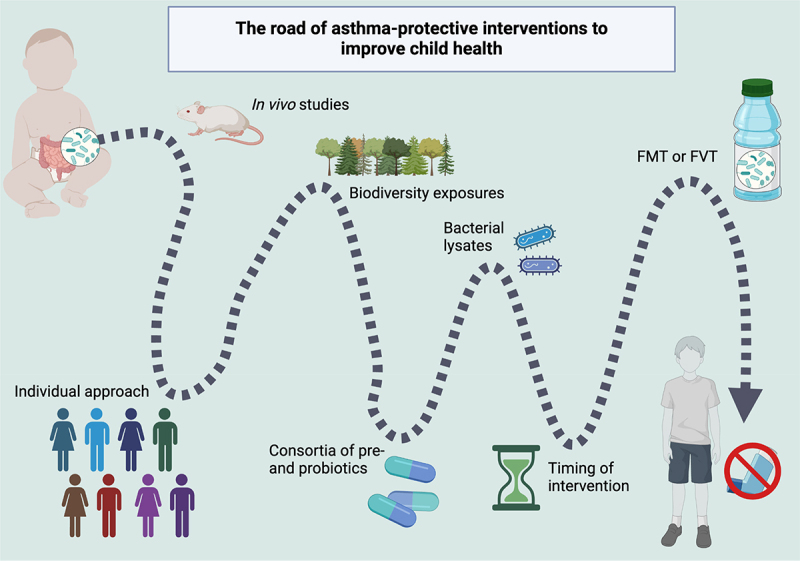
The road of asthma-protective interventions to improve child health.Here, we have summarized a few of the methods discussed in this review that are relevant to consider when designing intervention studies to decrease the risk of childhood asthma.

There is already a large amount of evidence gathered by association studies of the relevance of the gut microbiome in childhood asthma. However, these studies inherently confer little information on the underlying mechanisms or practical clinical applications. To build upon that evidence *in vivo* research on model organisms has shed some light on the underlying mechanisms. Furthermore, *in vivo* research can be useful for clinical de-risking and can create a bridge to decrease the disconnect between observational and intervention studies. Alongside this, the increasing amount of research focusing on human RCTs with various interventions has aimed at demonstrating the causal role of the gut microbiome in childhood asthma. However, there are conflicting findings, which is likely due to the complexities of the gut microbiome and the intricacies of childhood asthma that reduces the comparability between many intervention studies. We demonstrate in Figure 3 and summarize throughout this section, some considerations that we believe are important for future research to improve childhood asthma.

Future RCTs must be informed based on current knowledge, as many published RCTs are largely based on known probiotics from other diseases that may not translate well to early life or asthma etiology, leading to conflicting results that are not providing consistent evidence.

We believe that interventions such as FVTs, consortia of asthma-specific probiotic bacteria, or low-risk natural interventions, hold great potential in the future of RCTs in asthma. When considering consortia of bacteria, it is important that they are identified through previous asthma-specific research, and implemented in the specific population where they were first identified (e.g. children with genetic risk or to prevent only a specific asthma phenotype), likewise, their functional synergy should be evaluated to ensure complementary mechanisms. Additionally, we advise researchers implementing future intervention studies to focus on children at risk, both from microbial perturbations (e.g. early antibiotics and cesarean delivery), as well as host or environmental susceptibility (e.g. genetic risk or urban living), as the microbiome may be of particular importance here. Furthermore, due to the low complexity of the very early-life infant gut microbiome, interventions at this age are likely to yield highly influential and potentially long-lasting effects. Consistent monitoring throughout the first year of life is essential to evaluate microbial engraftment and clinical effect. After the cessation of breastfeeding and with the introduction of solid foods, the microbiome undergoes a large shift, which could also be capitalized for intervention. However, after approximately one year of age, the microbiome becomes more stable and similar to the adult gut, where the implementation of compositional changes may be challenging.

A general issue with microbiome RCTs is the lack of power, due to either low sample sizes, or difficulties with, e.g., analysis of a large number of samples, adherence to long supplementation protocols, or a long follow-up duration. With the cost of sequencing decreasing, and the increased understanding of the complexity of the human gut microbiome, there are ample opportunities to unveil the relationship of the gut microbes and asthma. To fully capitalize on this, researchers should harmonize protocols based on the chosen intervention type, carefully consider the biologically relevant timing and duration of the intervention and follow-up, and ensure that the number of participants meets appropriate power calculations based on the specific asthma outcome investigated. Furthermore, the clinical evaluations of asthma must also be harmonized to ensure that the same etiology is being studied and compared across studies. With an increased homogeneity across analysis methods and clinical evaluations, we foresee the gut microbiome as a key player in preventing asthma for children worldwide.

## Abbreviations

16S: 16S rRNA amplicon sequencing
AhR: Aryl hydrocarbon receptor
CHILD cohort study: Canadian Healthy Infant Longitudinal Development cohort study
COPSAC: Copenhagen prospective studies on asthma in childhood
DNA: Deoxyribonucleic acid
FFT: Fecal filtrate transfer
FLVR: Faecalibacterium, Lachnospira, Veillonella, and Rothia
FMT: Fecal microbial transplant
FVT: Fecal virome transplant
GPR: G-protein-coupled receptors
IDO: Indoleamine 2,3-dioxygenase
IFN-γ: Interferon-gamma
Ig: Immunoglobulin
IL: Interleukin
ISAAC: International Study of Asthma and Allergies in Childhood
MAZ: Microbiome-by-age z-score
NEC: Necrotizing enterocolitis
PASTURE birth cohort: Protection Against Allergy: Study in Rural Environments birth cohort study
RCT: Randomized control trial
SCFA: Short-chain fatty acid
TLR: Toll like receptor
VDAART: Vitamin D Antenatal Asthma Reduction Trial
